# The Impact of Oral Microbiome Dysbiosis on the Aetiology, Pathogenesis, and Development of Oral Cancer

**DOI:** 10.3390/cancers16172997

**Published:** 2024-08-28

**Authors:** Jasminka Talapko, Suzana Erić, Tomislav Meštrović, Marinka Mravak Stipetić, Martina Juzbašić, Darko Katalinić, Sanja Bekić, Dora Muršić, Josipa Flam, Dino Belić, Davor Lešić, Rajko Fureš, Manda Markanović, Ivana Škrlec

**Affiliations:** 1 Faculty of Dental Medicine and Health, Josip Juraj Strossmayer University of Osijek, 31000 Osijek, Croatiammstipetic@fdmz.hr (M.M.S.); dkatalinic@fdmz.hr (D.K.); dora.mursic@gmail.com (D.M.); rajko.fures@gmail.com (R.F.); 2 Department of Radiotherapy and Oncology, University Hospital Center Osijek, 31000 Osijek, Croatia; jflam@mefos.hr (J.F.);; 3 Faculty of Medicine, Josip Juraj Strossmayer University of Osijek, 31000 Osijek, Croatia; 4 University Centre Varaždin, University North, 42000 Varaždin, Croatia; tmestrovic@unin.hr; 5 Institute for Health Metrics and Evaluation, University of Washington, Seattle, WA 98195, USA; 6 Department for Health Metrics Sciences, School of Medicine, University of Washington, Seattle, WA 98195, USA; 7 Family Medicine Practice, 31000 Osijek, Croatia; 8 Fizio Educa, 31000 Osijek, Croatia; 9 Department of Gynecology and Obstetrics, Zabok General Hospital and Croatian Veterans Hospital, 49210 Zabok, Croatia; 10 Department of Clinical and Molecular Microbiology, University Hospital Centre Zagreb, 10000 Zagreb, Croatia; manda.markanovic@kbc-zagreb.hr

**Keywords:** microbiome, dysbiosis, pathobionts, inflammation, oral cancer, oral cavity

## Abstract

**Simple Summary:**

The problems associated with oral squamous cell carcinoma (OSCC) are becoming increasingly apparent. Namely, cancer of the oral cavity is the most common malignant tumor in the head and neck area worldwide. In addition to the usual risk factors for the development of OSCC, such as smoking and alcohol consumption, recent research has shifted its focus to the oral cavity microbiome, specifically dysbiosis. Numerous studies have concluded that disrupting the eubiosis of the oral cavity promotes the growth of oral pathogens, which, through their virulence factors and manifold pathogenicity factors, lead to inflammatory conditions that can damage tissue cells, potentially leading to cancer development. From an aetiological point of view, these pathogens can be bacteria, viruses, fungi, and parasites.

**Abstract:**

Oral squamous cell carcinoma (OSCC) is the most common head and neck cancer. Although the oral cavity is an easily accessible area for visual examination, the OSCC is more often detected at an advanced stage. The global prevalence of OSCC is around 6%, with increasing trends posing a significant health problem due to the increase in morbidity and mortality. The oral cavity microbiome has been the target of numerous studies, with findings highlighting the significant role of dysbiosis in developing OSCC. Dysbiosis can significantly increase pathobionts (bacteria, viruses, fungi, and parasites) that trigger inflammation through their virulence and pathogenicity factors. In contrast, chronic bacterial inflammation contributes to the development of OSCC. Pathobionts also have other effects, such as the impact on the immune system, which can alter immune responses and contribute to a pro-inflammatory environment. Poor oral hygiene and carbohydrate-rich foods can also increase the risk of developing oral cancer. The risk factors and mechanisms of OSCC development are not yet fully understood and remain a frequent research topic. For this reason, this narrative review concentrates on the issue of dysbiosis as the potential cause of OSCC, as well as the underlying mechanisms involved.

## 1. Introduction

One of the most common carcinomas of the head and neck is squamous cell carcinoma of the oral cavity [1,2]. The oral cavity extends anatomically from the vermilion border of the lips to the transition between the hard and soft palate or the posterior third of the tongue [3]; considering such complex anatomy and functional significance, malignancies in this area may significantly impact speech, swallowing, and the overall quality of life.

Potentially malignant disorders of the oral cavity, including leukoplakia and erythroplakia, carry a risk of developing into oral squamous cell carcinoma (OSCC). These conditions may present with various features, such as changes in mucosal coloration (red, white, or a combination of both), changes in the dimensions of the affected areas, as well as changes in morphology (smooth, ridged, granular, warty, thin, or plaque-like) [4,5,6]. Epithelial dysplasia in oral potentially malignant disease may depend on structural changes with little to no cellular abnormalities. While distinct features can be hard to recognize, the combination of molecular, clinical, and microscopic changes increases the risk of developing OSCC [4,7]. OSCC is an important head and neck cancer, occurring mainly in the lips, mouth, and oropharynx [8]. The oral cavity is a region that is easily accessible to the naked eye, but despite this, the cancer is more often detected at an advanced stage [9]. Squamous cell carcinoma of the oral cavity occurs annually in about 6% of the world’s population, 4% of which are men and 2% women [10]. In terms of mortality, it ranks 10th worldwide [11] and occurs most frequently after age 50 [12]. Oral and pharyngeal cancer represents a global public health problem. The annual incidence in 2020 was 377,713 cases, with half resulting in death [13]. The prevalence of oral cavity cancer is rising significantly on a global scale, particularly in the regions of Eastern and Western Europe, South Asia, the Caribbean, Latin America, and the Pacific [14]. India has the third-highest incidence of oral cancer in the world [15]. Similarly, the Republic of Croatia is experiencing an increasing incidence of oral cavity cancer, aligning with this global trend [16].

In the Republic of Croatia, 300–400 people die from oral cancer each year [16]. Squamous cell carcinoma of the tongue can be triggered by chronic irritants such as dental caries, dentures, poor oral hygiene, and excessive use of so-called mouthwashes [17], particularly those containing alcohol. Other risk factors include tobacco chewing and the use of betel, the latter being common in some parts of Asia and Africa. Human papillomavirus (HPV), transmitted orally via sexual contact, is also more frequently associated with oral cavity cancer and may contribute to its etiology [18,19]. Approximately 40% of squamous cell carcinomas of the oral cavity develop on the floor of the oral cavity and the lateral and ventral sides of the tongue [20]. Additionally, 38% of lower lip cancers are due to ultraviolet ray exposure [21]. Changes in the oral cavity are initially asymptomatic, making screening essential [22]. Hence, dentists should carefully examine the oral cavity and oropharynx during routine dental care and perform a cytological smear with a brush if suspicious lesions occur [23].

The lesions may be precancerous, leukoplakia, and erythroplakia, but can also be carcinomas in situ [24]. Exophytic lesions, which are often firmly indurated or ulcerated and have a hard and raised margin, are most likely carcinomas [25]. 

Pain is typically the leading symptom in this region, as are malpositioned teeth, swollen gingiva, and a stiff tongue [26]. An incisional or brush biopsy is required as the cytological smear is a landmark method to verify the disease [27]. Risk factors for oral cavity cancer are alcohol consumption, tobacco smoking, or a combination of those (95%) [28]. In recent years, oral cavity cancer research has focused on the oral cavity microbiome, i.e., dysbiosis [29]. Cancer is no exception among the systemic diseases influenced by the microbiome. Chronic infections are known to contribute to the development of cancer, with approximately 13% of the global cancer burden directly attributable to infectious agents [30]. 

In this review, we would like to present the current knowledge on the role of the oral microbiome, focusing on the role of bacteria, fungi, and viruses in developing oral cancer. We also aim to explore how dysbiosis and interactions among these microorganisms contribute to the development of OSCC. Additionally, we will discuss the mechanisms through which these microbial communities influence carcinogenesis in the oral cavity.

## 2. Microbiome of the Oral Cavity

The oral cavity hosts the second most diverse microbiome in the human body, following the intestinal microbiome, with approximately 700 species of bacteria identified in this area [31]. The group of oral bacteria consists of six main phyla—*Actinobacteria*, *Bacteroidetes*, *Firmicutes*, *Fusobacteria*, *Proteobacteria*, *and Spirochaetes*, and they account for 94% of the detected taxa [32]. The remaining 6% include *Chlamydia*, *Chlorobi*, *Chloroflexi*, *Gracilibacteria*, *Saccharibacteria*, *Synergistetes*, SR1, and *Tenericutes* [33]. The oral cavity provides an ideal environment for these bacteria, with saliva maintaining a pH of 6.5–7, favorable for their growth [34]. The average temperature of 37 °C also offers a stable habitat [35]. 

Table 1 lists Gram-positive and Gram-negative bacteria that are part of the microbiome of a healthy oral cavity. The genera *Streptococcus*, *Leptotrichia*, *Actinomyces*, *Neisseria*, *Peptostreptococcus*, *Fusobacterium*, and *Kingella* dominate among them [36].

**Figure 1:**
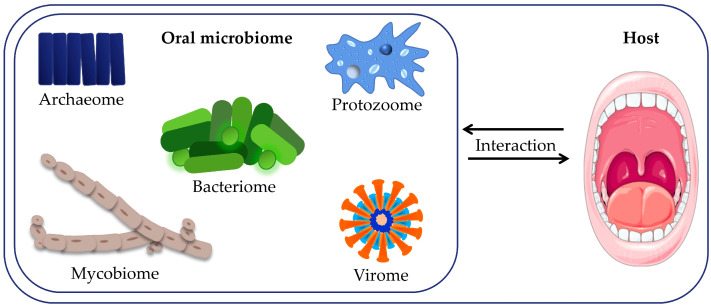
Interactions between the oral microbiome—the community of oral microorganisms consisting of numerous species of bacteria, fungi, viruses, archaea, and protozoa—living in the oral cavity and the host. Adapted and modified from articles [43,44] under the CC BY license.

Numerous microbial species that colonize the oral cavity are often found in eubiotics, i.e., in a harmonious coexistence that implies mutual interaction and interaction with their host, realized by very complex mechanisms [35] (Figure 1). Essentially, the development of the microbiome starts when the mother’s microbiome is transferred to the fetus through the mother’s saliva and the placenta, playing a crucial role in the development of the fetal immune system [37]. 

Colonization of the oral cavity begins very soon after birth, and the first colonizers are *Streptococcus mitis*, *Streptococcus sanguinis*, *Streptococcus gordonii*, and *Streptococcus salivarius* [38]. Until the first year of life, the oral cavity is primarily inhabited by aerobic bacteria, so in addition to streptococci, *Lactobacillus*, *Actinomyces*, *Neisseria*, and *Veillonella* are also present [39]. As all teeth emerge, the number of surfaces suitable for bacterial colonization increases [40]. Colonization with periodontal microbes results in gingival fissures, the formation of visible plaque in various parts of the tooth, and increased microbial diversity and succession [41]. The microbiome of the oral cavity changes and evolves throughout life, influenced by diet, hygiene habits, and host immunity. Over time, the community of microbial species is established, leading to a stable microbial environment in the oral cavity [38]. However, after all teeth are lost during aging, the microbiome reverts to a state similar to that before a child’s teeth emerge [42].

Identifying the microorganisms that colonize the oral cavity is challenging because bacteria from the environment (food, water, and air) rapidly enter the mouth. Additionally, colonization is influenced by contact between people, such as kissing [45]. Furthermore, assessment is complicated because most colonizers of the oral cavity are difficult to culture in laboratory conditions [31,46,47].

The most common identification tool to characterize the oral bacteriome is 16S rRNA amplicon sequencing using a target gene approach [48]. The highest concentration of microorganisms in the human body is found in saliva, oral mucosa, and dental plaque due to the abundance of nutrients necessary for their growth and multiplication [49]. 

Bacteria are the most predominant community of microorganisms in the oral microbiome [50]. The microbiome of the oral cavity also contains archaea [50]. All members of the archaeal community in the oral cavity obtain most of their metabolic energy through methane biosynthesis, which is why they are also referred to as methanogens [51]. Archaea are significantly more abundant in individuals suffering from periodontal disease, with *Methanobrevibacter oralis*, *Methanobacterium curvum*, *Methanobacterium congolense*, and *Methanosarcina mazeii* being the most common species [48]. In addition to them, there is also a mycobiome that includes various fungal communities [52]. *Candida* is the most abundant in the mycobiome, followed by *Cladosporium*, while *Aureobasidium* and *Saccharomycetales* account for about 50% of the oral mycobiome [53]. *Cryptococcus*, *Fusarium,* and *Aspergillus* are the least represented fungi [54]. 

In the dental mycobiome, 139 fungal species with 32 different taxa and one unclassified species of *Microdochium* with 12 taxa were documented. Analyses of the salivary mycobiome have identified two ecologically distinct ecotypes—the *Candida* monotype and the *Malassezia* monotype—which are salient biomarkers for oral diseases [55].

Many viruses coexist in the oral cavity, and most are considered pathogens. The microbiome’s viral component is called the virome [48]. It is regarded as a stable ecosystem that can infect host and bacterial cells, thus significantly affecting oral health [55]. Studies have shown an association between OSCC and oral viruses, namely Epstein–Barr virus (EBV), herpes simplex virus type 1 (HSV-1), hepatitis C virus (HCV), and HPV, and orphan viruses from the *Anelloviridae* family have also been detected [56]. Bacteriophages from class *Cudoviricetes* (myoviruses and siphoviruses) have been detected by analyzing the oral DNA virome [55].

It is known that the transformation of normal oral mucosa into OSCC occurs in several stages [57]. Potentially malignant disorders (OPMD) represent a high risk for malignant transformation. Indeed, it is known that about 80% of oral leukoplakia, proliferative verrucous leukoplakia, oral lichen planus (OLP), and oral submucous fibrosis (OSF) develop from these cases of oral cavity cancer [6,57]. The structure of the oral microbiome in oral cavity cancer differs from that of the normal oral mucosa, and in OPMD, the two overlap [58]. In patients with leukoplakia, colonization with *C. albicans* has been observed, which produces proteinase that degrades the basement membrane and produces carcinogens [43]. High levels of *Bacteroides* and *Fusobacteria* were also observed in this group of patients [59]. The phase of proliferative verrucous leukoplakia (PVL), which is a progressive form of multifocal leukoplakia with a significant rate of malignant transformation, differs significantly from that of healthy individuals. In patients with PVL, potentially antitumor pathogens such as *Oribacterium* sp., *Campylobacter jejuni*, *Eubacterium* sp., *Porphyromonas*, and *Tannerella* have been identified [60]. In patients with oral lichen planus (OLP), the number of *Porphyromonas* and *Solobacterium* is higher, and the number of *Haemophilus*, *Corynebacterium*, *Cellulosimicrobium* and *Campylobacter* is lower than in the healthy population [61]. In contrast, a higher prevalence of all HPV types has been found in oral submucosal fibrosis (OSF) than in the healthy population [62]. 

Chocolatewala et al. showed that the microbiome in saliva and tumor samples is not identical. *Exiguobacterium oxidotolerans*, *Prevotella melanogenic*, *Staphylococcus aureus*, and *Veillonella parvula* were isolated in the microbiome of tumor samples. In contrast, *Capnocytophaga gingivalis*, *Prevotella melanogenic*, and *Streptococcus mitis* were isolated from saliva samples associated with cancer [63].

### Role of Biofilm in the Development of Oral Cavity Cancer

It is important to note that oral cavity cancer is closely associated with the formation of biofilms [64]. Indeed, when bacterial biofilm growth occurs in the epithelium, potential conditions may lead to the oncogenic transformation of epithelial cells [65]. Recently, a frequent research target has been the biology of cancer, i.e., the study of the influence of bacterial biofilm on the cause itself and the progression of cancer [66]. 

The biofilm is essentially a sessile life form of bacteria [67], and biofilm formation is a dynamic and complex process in five phases [68]. The first phase is characterized by the adhesion and adherence of bacteria to surfaces in the oral cavity, whether they are soft tissues of the oral cavity, i.e., biotic surfaces, or dental implants, i.e., abiotic surfaces [69]. Bacterial species such as *Streptococcus* spp. are among the first colonizers. With the help of adhesins, they bind to specific receptors in the envelope, then multiply and secrete extracellular polymeric substances (EPS), which are important for the biofilm matrix [70]. Due to its bacillary form, *F. nucleatum* is an important bridge between initial colonizers such as *Streptococcus* spp. and later colonizers such as *P. gingivalis* [71]. Also, in inflammation, *F. nucleatum* plays a significant role in colorectal cancer [72]. 

At this stage, the lipopolysaccharides, fimbriae, and capsule that make up the surface structure of *P. gingivalis* and enable it to attach to the aforementioned surfaces are of exceptional importance [68,73]. *Actinomyces* spp. and *Veillonella* spp. belong to the secondary colonizers that join the primary biofilm colonizers by coaggregation [74]. In the second phase of biofilm formation, the biofilm matures, its architecture becomes increasingly complex, and it becomes a three-dimensional structure with microcolonies. These channels supply the cells with water, nutrients, and air [75]. Quorum sensing (QS) enables the control of bacterial population density with the help of extracellular molecular signals and autoinducers that control metabolism, biofilm production, and the occurrence of virulence [76]. The extracellular matrix consists of proteins, glycoproteins, extracellular polysaccharides, glycolipids, and nucleic acids [77]. The EPS matrix not only provides mechanical stability but also plays a key role in the function and structure of the biofilm and creates a complex chemical microenvironment necessary for the life of the biofilm [78]. In a mature dental biofilm, the dominant bacterial genera are *Actinomyces, Fusobacterium, Porphyromonas, Prevotella, Streptococcus,* and *Treponema*, which can vary greatly depending on the immune status of the host, diet, and oral hygiene [38]. In the last phase (dispersion phase), a transition from a sessile form to a mobile form takes place, in which the bacteria are released from the surface of the biofilm and have the opportunity to form new biofilms [79].

Biofilms are primarily associated with chronic infections. As they are also found in various types of cancer, numerous studies are being conducted to investigate their role in cancer development, progression, and treatment [80]. Recent research has shown that treatment outcomes and cancer behavior are significantly influenced by the microbial composition within the tumor microenvironment [81]. Biofilms are known to be resistant to the effects of antibiotics. They have very similar properties in cancer treatment [80]. Biofilms can essentially promote cancer through different mechanisms (Table 2).

## 3. Dysbiosis of the Oral Microbiota

**Figure 2:**
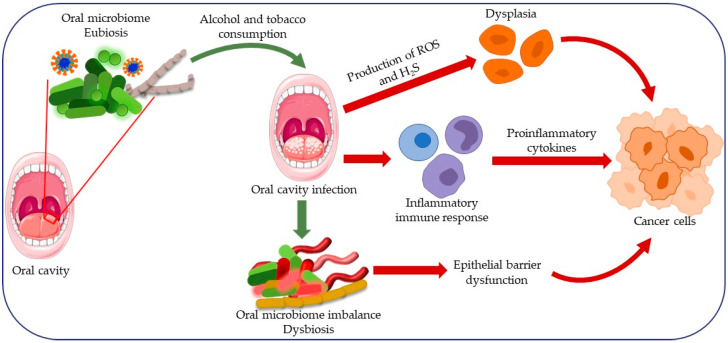
A depiction of the disruption of the balanced microbial community within the oral cavity (i.e., a dysbiosis of the oral microbiota) leading to carcinogenesis. Alcohol and tobacco have a negative effect on the composition of the oral microbiome and lead to dysbiosis. Oral infections caused by pathogenic bacteria like *Fusobacterium* and *Porphyromonas* species elevate cytokine levels and inflammatory factors. This results in chronic inflammation and changes in various molecular signaling pathways that regulate cell metabolism and growth. The substances produced by pathogenic bacteria, including ROS and H_2_S, induce genetic damage that promotes tumor development. Dysbiosis of the oral biofilm alters the homeostasis of the epithelial barrier and leads to barrier dysfunction. Adapted and modified from an article [43] under the CC BY license.

Microorganisms that are an integral part of the oral microbiome maintain a harmonious balance (homeostasis) of pH, nutrients, and temperature that are symbiotically or synergistically distributed [35]. However, changes caused by local or systemic factors can disrupt this balance, which we also refer to as “dysbiosis,” leading to the proliferation of potentially pathogenic microorganisms [36,85] (Figure 2). Dysbiosis of the oral microbiome can be triggered by excessive consumption of carbohydrate-rich foods, the breakdown of which creates an environment with a lower pH value. In such an environment, the growth of acid-sensitive microorganisms is inhibited, while microorganisms adapted to such an environment, such as *Lactobacillus* spp. and *Streptococcus mutants*, thrive [86]. This imbalance can lead to the development of numerous pathological conditions and diseases [87].

The presence of many pathogenic bacteria in the oral cavity can result in caries, gingivitis, and periodontal disease, leading to local inflammation [88]. Changes in the oral microbiota can influence OSCC through carcinogenic modulation of cell metabolism (e.g., by altering the concentration of vitamins and nutrients), thereby stimulating the production of various cytokines that may contribute to different pathological conditions [89]. OSCC can arise from chronic bacterial infection, and the link between chronic inflammation, periodontitis, and oral cancer is thought to be one of the leading causes [49]. Thus, it is important to note that dysbiosis disrupts the harmony in the microbiome of the oral cavity. When one microbial community is suppressed, another expands its pathogenic potential unhindered, which may be a significant factor in the development of OSCC [4,90]. Addressing and restoring microbial balance in the oral cavity could be a pivotal strategy in preventing and managing OSCC. 

Bacteria of the oral microbiome can contribute to oral carcinogenesis by various mechanisms, e.g., by activating cell proliferation, inhibiting apoptosis, triggering chronic inflammation, stimulating cell invasion, and producing carcinogens [91].

### 3.1. Bacteria Essential for the Development of Oral Cavity Cancer

Several studies have confirmed the close relationship between oral bacteria and OSCC. This process is greatly facilitated by the possibility of sequencing 16S rRNA amplicons to compare oral bacterial DNA isolated from cancer patients and healthy individuals [92,93,94]. Oral bacteria play an essential role in the development of OSCC. Thus, it is crucial to acknowledge the mechanisms that influence the development of OSCC, such as inhibition of apoptosis, acceleration of cell proliferation, and enhancement of tumor invasion and metastasis [95]. Understanding these mechanisms provides valuable insights into new diagnostic and treatment options for OSCC [96]. Particular attention should be given to pathogenic periodontal bacteria, which are linked to periodontal disease and contribute significantly to the inflammatory state that can induce DNA damage in epithelial cells, thereby promoting cancer progression [97].

*Fusobacterium nucleatum* (*F. nucleatum*) is an anaerobic, Gram-negative bacterium commonly found in the oral cavity [71]. Recent research has emphasized its important role in the pathogenesis of oral cavity cancer, particularly OSCC [98]. The association between *F. nucleatum* and oral cavity cancer is mediated through several mechanisms, including modulation of the immune response, promotion of inflammation, and direct interaction with cancer cells [99]. This bacterial agent can induce chronic inflammation in the oral cavity, a known risk factor for carcinogenesis [100]. *F. nucleatum* stimulates the production of pro-inflammatory cytokines such as IL-6, IL-8, and TNF-α., creating a microenvironment that favors cancer development by promoting cell proliferation, inhibiting apoptosis, and inducing DNA damage [101]. Additionally, it modulates the host immune response to promote tumor growth, inhibiting the activity of natural killer (NK) cells and cytotoxic T lymphocytes and allowing, in turn, cancer cells to evade immune surveillance [102]. 

*F. nucleatum* also promotes the recruitment of myeloid-derived suppressor cells (MDSCs) and regulatory T cells (Tregs), further suppressing the antitumor immune response [103]. *F. nucleatum* possesses adhesins such as *Fusobacterium* adhesin A (FadA), which facilitate its attachment to epithelial cells and extracellular matrix components. FadA can bind to E-cadherin on host cells, activating β-catenin signaling pathways that promote cell proliferation and survival [104]. This interaction supports bacterial invasion and contributes to the malignant transformation of epithelial cells [105]. Furthermore, this species can produce various metabolites and enzymes that can have genotoxic effects on host cells [106]; for instance, hydrogen sulfide (H_2_S), a metabolic byproduct of *F. nucleatum*, can cause DNA damage and mutations. Producing reactive oxygen species (ROS) by the bacterium exacerbates oxidative stress and genetic instability, which are key carcinogenic factors [107]. The presence of *F. nucleatum* in oral cancer tissues and saliva has been suggested as a potential biomarker for the early detection and prognosis of OSCC [108]. Quantitative PCR and next-generation sequencing techniques can detect and quantify *F. nucleatum* in clinical samples, helping to identify high-risk individuals [109]. 

*Porphyromonas gingivalis (P. gingivalis*) is a Gram-negative, anaerobic bacterium best known for its role in periodontal disease [68]. Recent findings suggest that *P. gingivalis*, similar to *F. nucleatum*, is involved in the development of oral cavity cancer, particularly oral squamous cell carcinoma [100]. The bacterium contributes to carcinogenesis via several mechanisms, including chronic inflammation, immunomodulation, direct interactions with epithelial cells, and manipulation of the tumor microenvironment [110]. This bacterium triggers a chronic inflammatory reaction in the oral cavity, a significant risk factor for cancer development [111]. 

This bacterial agent stimulates the production of pro-inflammatory cytokines such as IL-1β, IL-6, IL-8, and TNF-α by gingival epithelial cells and macrophages [112]. The cytokines above create a pro-tumourigenic environment by promoting cell proliferation, survival, and angiogenesis and inhibiting apoptosis [113]. *P. gingivalis* modulates the host immune response to evade detection and facilitate chronic infection [114]. It inhibits neutrophil and macrophage function, preventing adequate bacterial clearance. It also manipulates dendritic cell function, resulting in impaired antigen presentation and a biased T-cell response favoring a Th2 and Treg profile over a Th1 profile, which is less effective in antitumor immunity [115]. This immune modulation supports a microenvironment that allows cancer cells to proliferate and evade immune surveillance [116]. Several virulence factors, including fimbriae and gingipains, facilitate adhesion to and invasion of epithelial cells [114]. Gingipains are cysteine proteinases that belong to the peptidase family C25 [117]. The bacterium can invade oral epithelial cells and persist intracellularly, leading to changes in cell signaling pathways [118]. *P. gingivalis* can activate the NF-κB and MAPK signaling pathways involved in inflammation, cell survival, and proliferation [119]. By inhibiting apoptosis, it may confer a survival advantage to both the bacterium and the infected host cell, which may accumulate mutations and become malignant over time. Epithelial-mesenchymal transition (EMT) induced by *P. gingivalis* is mediated by activation of TGF-β and Wnt signaling pathways, resulting in increased expression of mesenchymal markers such as N-cadherin and vimentin, as well as decreased expression of epithelial markers such as E-cadherin [120]. 

The synergy between *P. gingivalis* and other microorganisms, such as *F. nucleatum*, enhances their pathogenicity and ability to promote a pro-inflammatory and pro-carcinogenic environment [121] (Table 3). Biofilms formed on the oral surface provide a reservoir for these bacteria, maintaining chronic inflammation and constant exposure of epithelial cells to bacterial virulence factors [122]. Preserving oral hygiene and reducing exposure to *F. nucleatum* and *P. gingivalis* through regular dental care and antimicrobial mouth rinses can reduce the risk of oral cancer [123]. Public health initiatives that promote oral health awareness and regular dental check-ups are vital in preventing bacterial colonization and its harmful effects [124]. Antimicrobial mouthwashes and professional dental cleanings can help manage bacterial populations in the oral cavity [125].

According to published studies, *Aggregatibacter actinomycetemcomitans* (*A. actinomycetemcomitans*) is directly involved in the development of OSCC [127]. Its oncogenic potential is directly related to its virulence factors (such as cytolethal toxin), which induce other byproducts like acetaldehyde, ROS, sulfides, nitrosamines, and reactive nitrogen species [30]. They collectively exhibit an oncogenic potential characterized by DNA alkylation, mutations, and significantly impaired repair [30]. Since *A. actinomycetemcomitans* stimulates the production of immunosuppressive cytokines such as IL-10, which can inhibit antitumor immunity, it is reasonable to conclude that it slows down the host immune response in a way that may stimulate and facilitate tumor growth [127]. 

### 3.2. Viral Causes of Oral Cavity Carcinoma

As already mentioned, oral cavity carcinoma, a type of head and neck cancer, is a major global health problem with rising incidence rates worldwide [128]. While traditional risk factors, such as tobacco use and alcohol consumption, continue to play a pivotal role, the role of viral infections in the etiology of oral cavity cancer has gained considerable attention in recent years [129]. While HPV infection remains the best-characterized and well-known viral cause of oral cavity carcinoma, EBV and Human herpesvirus 8 (HHV-8) infections highlight the diversity of viruses that can contribute to oral carcinogenesis [130]. Understanding the immunological responses to these viral infections provides valuable insights into tumor progression and offers promising opportunities for targeted therapies and immunotherapeutic approaches [131].

#### 3.2.1. Human Papillomavirus (HPV)

Human papillomavirus is a critical etiologic factor in oral cavity cancers, particularly in cases not associated with traditional risk factors [132]. HPV, most notably high-risk types such as HPV-16 and HPV-18, are repeatedly associated with the development of oropharyngeal cancer [133,134]. The viral oncoproteins E6 and E7 are primarily involved in HPV-induced carcinogenesis [135,136]. These proteins interfere with critical cellular metabolic pathways; more specifically, the E6 protein targets the degradation of the tumor suppressor p53, thus impairing cell cycle arrest and apoptosis, while the E7 protein inactivates the retinoblastoma protein (pRb), which leads to uncontrolled cell proliferation [137].

The host’s immune response to HPV infection plays a decisive role in the outcome of the viral infection and the possible development of cancer [138]. HPV infection triggers the innate immune response [139], with keratinocytes producing interferons and pro-inflammatory cytokines to activate NK cells and dendritic cells [140]. The adaptive immune response, especially CD4+ and CD8+ T cells, is essential for clearing HPV-infected cells [141]. However, HPV employs various mechanisms to evade immune recognition, including downregulating MHC class I molecules and interfering with interferon signaling [3]. Recent studies indicate that HPV-positive oral cavity carcinomas often exhibit a different immune microenvironment compared to HPV-negative tumors, with increased infiltration of CD8+ T cells and a better prognosis [142].

#### 3.2.2. Epstein-Barr Virus (EBV)

Epstein-Barr virus (EBV), a member of the herpesvirus family, is associated with the development of various malignancies, including nasopharyngeal carcinomas and a subset of oral cavity carcinomas [143]. Multiple viral proteins are involved in EBV-induced carcinogenesis. Latent membrane protein 1 (LMP1) acts as a constitutively active tumor necrosis factor receptor, activating the NF-κB and MAPK pathway to promote cell survival and proliferation [144]. Epstein-Barr nuclear antigen 1 (EBNA1) maintains the viral genome and interferes with the p53 and pRb signaling pathways [145]. The immune response to EBV in the context of oral cavity carcinoma is complex. Triggered by EBV infection, innate immunity produces type I interferons and activates NK cells [146]. EBV-specific T cells play a crucial role in the control of viral replication. However, EBV employs various strategies to evade the adaptive immune response, including the expression of viral IL-10, which suppresses the T-cell response [147]. Immunotherapies targeting EBV help treat EBV-associated oral cancers by enhancing T-cell responses against viral antigens [148,149].

#### 3.2.3. Human Herpesvirus 8 (HHV-8)

Human herpesvirus 8 (HHV-8), also known as Kaposi’s sarcoma-associated herpesvirus (KSHV), has been identified in a small subset of oral cavity carcinomas, particularly in immunocompromised individuals [150,151]. Several viral proteins contribute to the oncogenesis of HHV-8 [152,153]. There is a latency-associated nuclear antigen (LANA) that inhibits p53 and pRb, thereby promoting cell cycle progression, but also a viral G protein-coupled receptor (vGPCR) that activates several signaling pathways (including PI3K/AKT and MAPK), thereby promoting angiogenesis and cell survival [154]. The immune response to HHV-8 in oral cavity carcinoma is less well understood compared to HPV and EBV. However, studies have shown that HHV-8 infection activates pattern recognition receptors that produce type I interferons and that CD8+ T cells specific for HHV-8 antigens play a critical role in controlling viral replication [155]. Akin to other viruses, HHV-8 employs various strategies to evade the immune system, including the expression of viral proteins that interfere with antigen presentation and T-cell activation [156].

#### 3.2.4. Immunologic Microenvironment and Therapeutic Implications

The immunological microenvironment of virus-associated oral cavity carcinomas plays a crucial role in tumor progression and response to therapy [157]. These cancers often exhibit increased expression of immune checkpoint molecules such as PD-L1, which are potential targets for immunotherapy [158]. The presence and composition of tumor-infiltrating lymphocytes (TILs) in virus-associated oral cavity cancers have been shown to have prognostic significance, with higher levels of CD8+ T cells generally linked to better outcomes [159,160]. Virus-associated oral cavity cancers also frequently show a distinct cytokine profile with elevated levels of pro-inflammatory cytokines such as IL-6 and TNF-α, which may influence tumor progression and response to treatment [161,162].

Understanding the complex interplay between viral infection, host immune response, and tumor microenvironment has led to the development of new therapeutic approaches and strategies [163,164]. Ongoing clinical trials evaluate the efficacy of therapeutic vaccines targeting viral antigens in HPV- and EBV-associated oral cavity cancers [164]. Immune checkpoint inhibitors, such as pembrolizumab and nivolumab, have shown promising results in the treatment of recurrent or metastatic squamous cell carcinoma of the head and neck, including virus-associated cases [165]. Adoptive T-cell therapy targeting viral antigens is being explored as a potential treatment option for virus-associated oral cavity cancers [166]. These advancements highlight the importance of personalized immunotherapy in improving patient outcomes.

### 3.3. Role of Fungi in Oral Carcinoma

After a significant increase in the incidence of certain fungi was observed in cancer patients, the connection between fungi and various malignancies in humans has gained increasing attention in recent years and sparked extensive research efforts to understand the underlying mechanisms [167].

More than 75 genera of fungi, including *Candida*, *Cladosporium*, *Aureobasidium*, and *Aspergillus*, comprise the diverse fungal community in the oral cavity. Fungal infections, particularly those caused by *Candida* species, have been associated with the pathogenesis of oral carcinoma (OC) [4,168]. Fungi can increase cell density and stimulate hyphal growth, providing the structural basis for biofilms with various pathogens. Furthermore, among eukaryotes, fungi are notable for their significant impact on the host immune system and a plethora of immunological effects. The interactions between host and fungi highlight a robust immune response mechanism in the host [169,170]. Fungal infections can cause chronic inflammation in the oral cavity, which is a known risk factor for the development of various cancers, including OC. Persistent inflammation can result in cellular damage, DNA mutations, and an environment conducive to malignant transformation [4].

Metagenomic analysis of whole-genome sequencing (WGS) data revealed an association between *C. albicans* and head and neck tumors. Using Illumina™ 2 × 300 bp chemistry, *C. albicans* was found to play a role in initiating and developing OC based on its ITS2 region [167]. *C. albicans* promotes OC via IL-17A/IL-17RA and macrophage involvement [171,172]; more specifically, the infection with *C. albicans* increases the production of IL-17A by Th17 cells [173]. Tumor cells can release the chemokine (C-C motif) ligand 2 (CCL2) upon activating IL-17RA signaling to attract macrophages into the tumor microenvironment. These macrophages exhibit an immunosuppressive phenotype with increased expression of IL-10, arginase-1, PD-L1, and galectin-9 [167].

*Candida* can also interact with other microbial pathogens in the oral cavity to form biofilms. These biofilms create a complex and protective environment that enhances the pathogenic potential of the involved microorganisms. Such an interaction leads to increased inflammation and tissue damage, providing a persistent inflammatory stimulus that further promotes carcinogenesis [174,175].

It is important to note that *C. albicans* plays a vital role in metabolic interactions related to oxygen removal, creating a favorable niche for the survival of *P. gingivalis* in the oral cavity [176]. Similarly, several oral bacteria physically interact with the hyphae of *C. albicans* [175], with specific surface proteins associated with the hyphae playing an important role. These are members of the Als1 and Als3 families of agglutinin-like sequences (Als), the hyphal wall protein 1 (Hwp1), and the cell wall adhesion protein 1 (Eap1) [177,178]. Both metabolic and physical interactions between *P. gingivalis* and *C. albicans* can enhance the invasive capability of *P. gingivalis* [176]. Interactions between *C. albicans* and *P. gingivalis* show that cohesion induced by specific proteins causes significant changes in the gene expression of *P. gingivalis*, which can lead to increased infectivity [176,179].

A group of authors investigated the correlation between fungi and bacteria in 39 specimens, including non-tumorous and tumorous tongue samples. The fungal species *Lichtheimia corymbifera* showed a positive association with bacterial genera such as *Fusobacterium*, *Porphyromonas*, and *Campylobacter* [180]. This suggests that interactions between different groups of microorganisms can cause specific oral diseases, including precancerous lesions [35], underscoring the complex interplay within the oral microbiome that may drive the progression of these conditions.

*C. albicans* is the most common fungal species found in the oral cavity and has been extensively studied in the context of OC. This yeast can produce nitrosamines and metabolize ethanol to acetaldehyde, an electrophilic and genotoxic substance that impacts DNA repair, induces oxidative stress, and causes DNA damage. These byproducts can cause mutations in the epithelial cells of the oral mucosa, potentially resulting in malignant transformation [43,181,182].

Virulence factors, lipolytic activity, and the ability of *C. albicans* to degrade proteins considerably influence the development of carcinogenesis. At the same time, further invasion into the tissue is favored by hydrolytic exoenzymes [182,183]. Proteinase, phospholipase, and *C. albicans* lipase activity are notably higher in patients with OC [184]. Candidalysin, a cytolytic toxin, is the most important virulence factor of *C. albicans* [185,186], playing a crucial role in the induction of cell damage and inflammation [187]. The inflammatory molecules IL-6, IL-17, NLRP3, and GM-CSF are associated with carcinogenesis [188]. Candidalysin is encoded by the ECE1 gene, which is associated with *C. albicans* virulence factors such as adhesion, filamentation, and biofilm formation [189]. *C. albicans* biofilm may contribute to the development and progression of OC by inducing the formation of lipid droplets and reducing the efficacy of chemotherapeutic agents [190]. Similarly, candidalysin can stimulate signaling pathways critical in carcinogenesis [181]. Candidalysin also promotes angiogenesis and the formation of new blood vessels, which are essential for cancer growth and metastasis of primary tumors to other tissues and organs—suggesting its significant role in the progression of oral cavity cancer [43]. 

Individuals with weakened immune systems, such as people with human immunodeficiency virus/acquired immunodeficiency syndrome (HIV/AIDS), diabetes, or those undergoing immunosuppressive therapy (e.g., corticosteroids, chemotherapy), are more prone to fungal infections. These infections can elevate the risk of developing oral carcinoma as the body is less capable of combating abnormal cell growth [43,191]. 

Oral infections caused by *Candida species* lead to an upregulation of pro-inflammatory cytokines, including interleukin (IL)-1α, IL-1β, IL-6, IL-8, IL-18, tumor necrosis factor (TNF)-α, IFN-γ and GM-CSF. These cytokines directly impact metabolic pathways and, accordingly, induce endothelial dysfunction, playing an essential role in mechanisms related to the immune system and cancer development [192].

Fungal infections can also stimulate the proliferation of epithelial cells in the oral cavity. This increased cell turnover heightens the likelihood of genetic mutations and abnormal cell growth, setting the stage for cancer development [4,193,194]. Although fungal infections alone are insufficient to cause oral carcinoma, they play an important role in creating a microenvironment that promotes carcinogenesis through chronic inflammation, molecular changes, and interactions with other pathogenic microbial species. Effective treatment of fungal infections and oral hygiene maintenance are essential strategies to reduce the risk of oral carcinoma, as well as to prevent the progression of potentially malignant conditions in the oral cavity.

### 3.4. Role of Protozoa in Oral Carcinoma

Oral parasites in the oral microbiome include protozoa, typically found in periodontal pockets and dental plaque [36]. *Entamoeba gingivalis* (*E. gingivalis*) and *Trichomonas tenax* (*T. tenax*) are oral protozoan parasites commonly seen in patients with poor oral hygiene and chronic and periodontal diseases. The transmission of *T. tenax* is possible through contaminated water, food, and saliva, with prevalence in the mouth ranging from 4% to 53% [195]. Research by Malaa and colleagues found that the prevalence of *E. gingivalis* or *T. tenax* varies depending on the metabolic disorder, indicating a potential link between specific metabolic conditions and the susceptibility to these oral protozoan parasites [195] (Table 4).

Although parasites are present in low numbers in the oral cavity, recent studies using microbial biomarkers for human OSCC tissue have also revealed oncogenic parasites. However, further studies are needed to confirm this and draw a definitive conclusion [196].

## 4. Conclusions

The microbiota of the oral cavity consists of various microorganisms, including bacteria, viruses, protozoa, fungi, and archaea. This diverse microbial community plays an extremely important role in the activities of the immune system and the metabolism of various substances. Dysbiosis, a disruption in the normal composition of the microbiome, can lead to chronic inflammation, which significantly contributes to the development of OSCC. To prevent OSCC, a careful examination of the microbiota of the oral cavity and increased oral hygiene measures are steps of utmost importance. This involves routine dental check-ups and the use of antimicrobial mouthwashes and professional cleanings to manage and maintain a healthy balance of microorganisms in the oral cavity.

Furthermore, a deeper investigation into the influence of the microbiota on the pathogenesis of OSCC is necessary. We showed some notable advancement in the field, but fully understanding the specific mechanisms by which dysbiosis promotes carcinogenesis will undoubtedly aid in the discovery of potential therapeutic targets. For instance, targeting the pro-inflammatory pathways activated by pathogenic microorganisms or developing probiotics that can restore a healthy microbiome balance are just two examples of rather promising future strategies. Ultimately, fostering a comprehensive approach to oral health and microbiome research will be pivotal in our quest to combat OSCC and improve patient outcomes.

## Figures and Tables

**Table 1:** Genera of Gram-positive and Gram-negative bacteria that make up the microbiome of a healthy oral cavity.

Gram + Cocci	Gram + Bacilli	Gram − Cocci	Gram − Bacilli
*Abiotrophia* *Peptostreptococcus* *Streptococcus*	*Actinomyces*	*Moraxella*	*Campylobacter*
	*Bifidobacterium*	*Neisseria*	*Capnocytophaga*
	*Corynebacterium*	*Veillonella*	*Desulfobacter*
*Stomatococcus*	*Eubacterium*		*Desulfovibrio*
	*Lactobacillus*		*Eikenella*
	*Propionibacterium*		*Fusobacterium*
	*Pseudoramibacter*		*Hemophilus*
	*Rothia*		*Leptotrichia*
			*Prevotella*
			*Selemonas*
			*Simonsiella*
			*Treponema*
			*Wolinella*

**Table 2:** Mechanisms by which biofilm promotes the formation of cancer.

No.	Mechanisms by Which Biofilm Promotes the Formation of Cancer	Ref.
1.	Biofilm can directly affect the immune response of the host, thus creating a favorable environment for the development of cancer.	[66]
2.	It can trigger chronic inflammation that leads to DNA damage and thus promotes the growth of cancer cells.	[82]
3.	Bacteria inside the biofilm can secrete toxins that have carcinogenic effects.	[83]
4.	Bacteria found in the tumor microenvironment (tumor microbiome) influence cancer progression.	[84]
5.	Bacteria in the biofilm can significantly change the metabolism of the host.	[70]

**Table 3:** A summary of the oncogenic effects described for the bacteria *F. nucleatum* and *P. gingivalis*.

Bacteria	Oncogenic Effects	Ref.
*F. nucleatum* *P. gingivalis*	Inducing infected cells to produce inflammatory cytokines or growth factors Induction of epithelial-to-mesenchymal transition Increase in proliferation Establishment of a tumor-promoting immune environment induction of chemoresistance, Induction of epithelial-to-mesenchymal transition Release of mutagenic substances Upregulation of cell survival factors Stimulation of cell invasion Suppression of antitumor immune response Initiation of tumor angiogenesis Enhancement of cellular stemness	[99,126]

**Table 4:** Representation of parasites in the oral cavity depending on metabolic disorders.

Parasite	Representation of Parasites in the Oral Cavity
*Entamoeba gingivalis*	Highly prevalent in people with diabetes
*Trichomonas tenax*	Low in thyroid disorders Highly prevalent in people with hypertension

## Data Availability

Not applicable.

## References

[1] Johnson D.E., Burtness B., Leemans C.R., Lui V.W.Y., Bauman J.E., Grandis J.R. (2020). Head and Neck Squamous Cell Carcinoma. Nat. Rev. Dis. Primers.

[2] Badwelan M., Muaddi H., Ahmed A., Lee K.T., Tran S.D. (2023). Oral Squamous Cell Carcinoma and Concomitant Primary Tumors, What Do We Know? A Review of the Literature. Curr. Oncol..

[3] Montero P.H., Patel S.G. (2015). Cancer of the Oral Cavity. Surg. Oncol. Clin. N. Am..

[4] Monteiro J.S., Kaushik K., de Arruda J.A.A., Georgakopoulou E., Vieira A.T., Silva T.A., Devadiga D., Anyanechi C.E., Shetty S. (2024). Fungal Footprints in Oral Cancer: Unveiling the Oral Mycobiome. Front. Oral Health.

[5] Farah C.S., Woo S.B., Zain R.B., Sklavounou A., McCullough M.J., Lingen M. (2014). Oral Cancer and Oral Potentially Malignant Disorders. Int. J. Dent..

[6] Kumari P., Debta P., Dixit A. (2022). Oral Potentially Malignant Disorders: Etiology, Pathogenesis, and Transformation Into Oral Cancer. Front. Pharmacol..

[7] Ranganathan K., Kavitha L. (2019). Oral Epithelial Dysplasia: Classifications and Clinical Relevance in Risk Assessment of Oral Potentially Malignant Disorders. J. Oral Maxillofac. Pathol..

[8] Farooq I., Bugshan A. (2020). Oral Squamous Cell Carcinoma: Metastasis, Potentially Associated Malignant Disorders, Etiology and Recent Advancements in Diagnosis. F1000Research.

[9] Romano A., Di Stasio D., Petruzzi M., Fiori F., Lajolo C., Santarelli A., Lucchese A., Serpico R., Contaldo M. (2021). Noninvasive Imaging Methods to Improve the Diagnosis of Oral Carcinoma and Its Precursors: State of the Art and Proposal of a Three-Step Diagnostic Process. Cancers.

[10] Haj-Hosseini N., Lindblad J., Hasséus B., Kumar V.V., Subramaniam N., Hirsch J.M. (2024). Early Detection of Oral Potentially Malignant Disorders: A Review on Prospective Screening Methods with Regard to Global Challenges. J. Maxillofac. Oral Surg..

[11] Bray F., Laversanne M., Sung H., Ferlay J., Siegel R.L., Soerjomataram I., Jemal A. (2024). Global Cancer Statistics 2022: GLOBOCAN Estimates of Incidence and Mortality Worldwide for 36 Cancers in 185 Countries. CA Cancer J. Clin..

[12] Ferreira e Costa R., Leão M.L.B., Sant’Ana M.S.P., Mesquita R.A., Gomez R.S., Santos-Silva A.R., Khurram S.A., Tailor A., Schouwstra C.M., Robinson L. (2022). Oral Squamous Cell Carcinoma Frequency in Young Patients from Referral Centers Around the World. Head Neck Pathol..

[13] Tranby E.P., Heaton L.J., Tomar S.L., Kelly A.L., Fager G.L., Backley M., Frantsve-Hawley J. (2022). Oral Cancer Prevalence, Mortality, and Costs in Medicaid and Commercial Insurance Claims Data. Cancer Epidemiol. Biomark. Prev..

[14] Sung H., Ferlay J., Siegel R.L., Laversanne M., Soerjomataram I., Jemal A., Bray F. (2021). Global Cancer Statistics 2020: GLOBOCAN Estimates of Incidence and Mortality Worldwide for 36 Cancers in 185 Countries. CA Cancer J. Clin..

[15] Asmin P.K., Nusrath F., Divakar D.D. (2024). Occurrence and Distribution of Cancers with Emphasis Upon Oral Cancers in Registered Oncology Institutes of South India—A Retrospective Study. Indian J. Community Med..

[16] Cigic L., Martinovic D., Martinic J., Kovic M., Druzijanic A., Galic I., Tadin A., Lukanovic B., Duzel M., Poklepovic-Pericic T. (2023). Increased Prevalence of Oral Potentially Malignant Lesions among Croatian War Invalids, a Cross-Sectional Study. J. Clin. Exp. Dent..

[17] Girisa S., Kumar A., Rana V., Parama D., Daimary U.D., Warnakulasuriya S., Kumar A.P., Kunnumakkara A.B. (2021). From Simple Mouth Cavities to Complex Oral Mucosal Disorders-Curcuminoids as a Promising Therapeutic Approach. ACS Pharmacol. Transl. Sci..

[18] Wierzbicka M., San Giorgi M.R.M., Dikkers F.G. (2023). Transmission and Clearance of Human Papillomavirus Infection in the Oral Cavity and Its Role in Oropharyngeal Carcinoma—A Review. Rev. Med. Virol..

[19] Dellino M., Pinto G., D’Amato A., Barbara F., Di Gennaro F., Saracino A., Laganà A.S., Vimercati A., Malvasi A., Malvasi V.M. (2024). Analogies between HPV Behavior in Oral and Vaginal Cavity: Narrative Review on the Current Evidence in the Literature. J. Clin. Med..

[20] Mohideen K., Krithika C., Jeddy N., Bharathi R., Thayumanavan B., Sankari S.L. (2019). Meta-Analysis on Risk Factors of Squamous Cell Carcinoma of the Tongue in Young Adults. J. Oral Maxillofac. Pathol..

[21] Zhang J., Yang Q., Wu J., Yuan R., Zhao X., Li Y., Cheng X., Wu B., Zhu N. (2023). Trends in Cutaneous Squamous Cell Carcinoma on the Lip Incidence and Mortality in the United States, 2000–2019. Front. Oncol..

[22] Warnakulasuriya S., Kerr A.R. (2021). Oral Cancer Screening: Past, Present, and Future. J. Dent. Res..

[23] Neumann F.W., Neumann H., Spieth S., Remmerbach T.W. (2022). Retrospective Evaluation of the Oral Brush Biopsy in Daily Dental Routine—An Effective Way of Early Cancer Detection. Clin. Oral Investig..

[24] Deshmukh V., Shekar K. (2021). Oral Squamous Cell Carcinoma: Diagnosis and Treatment Planning. Oral and Maxillofacial Surgery for the Clinician.

[25] Mortazavi H., Safi Y., Baharvand M., Rahmani S., Jafari S. (2017). Peripheral Exophytic Oral Lesions: A Clinical Decision Tree. Int. J. Dent..

[26] Öhman J., Zlotogorski-Hurvitz A., Dobriyan A., Reiter S., Vered M., Willberg J., Lajolo C., Siponen M. (2023). Oral Erythroplakia and Oral Erythroplakia-like Oral Squamous Cell Carcinoma—What’s the Difference?. BMC Oral Health.

[27] Alwahaibi N., Alghallabi A., Alsinawi S., Aldairi N. (2018). Cytological Smear and Cell Block Versus Tissue Biopsies in the Diagnosis of Malignant Tumours in Non-Gynaecologic Specimens. Ethiop. J. Health Sci..

[28] Edirisinghe S.T., Devmini T., Pathmaperuma S., Weerasekera M., De Silva K., Liyanage I., Niluka M., Madushika K., Deegodagamage S., Wijesundara C. (2023). Risk Assessment of Alcohol Consumption for Oral Cancer: A Case-Control Study in Patients Attending the National Cancer Institute (Apeksha Hospital, Maharagama) of Sri Lanka. Asian Pac. J. Cancer Prev..

[29] Unlu O., Demirci M., Paksoy T., Eden A.B., Tansuker H.D., Dalmizrak A., Aktan C., Senel F., Sunter A.V., Yigit O. (2024). Oral Microbial Dysbiosis in Patients with Oral Cavity Cancers. Clin. Oral Investig..

[30] Irfan M., Delgado R.Z.R., Frias-Lopez J. (2020). The Oral Microbiome and Cancer. Front. Immunol..

[31] Deo P.N., Deshmukh R. (2019). Oral Microbiome: Unveiling the Fundamentals. J. Oral Maxillofac. Pathol..

[32] Dong L., Yin J., Zhao J., Ma S.R., Wang H.R., Wang M., Chen W., Wei W.Q. (2018). Microbial Similarity and Preference for Specific Sites in Healthy Oral Cavity and Esophagus. Front. Microbiol..

[33] Santonocito S., Giudice A., Polizzi A., Troiano G., Merlo E.M., Sclafani R., Grosso G., Isola G. (2022). A Cross-Talk between Diet and the Oral Microbiome: Balance of Nutrition on Inflammation and Immune System’s Response during Periodontitis. Nutrients.

[34] Lee Y.H., Chung S.W., Auh Q.S., Hong S.J., Lee Y.A., Jung J., Lee G.J., Park H.J., Shin S., Hong J.Y. (2021). Progress in Oral Microbiome Related to Oral and Systemic Diseases: An Update. Diagnostics.

[35] Santacroce L., Passarelli P.C., Azzolino D., Bottalico L., Charitos I.A., Cazzolla A.P., Colella M., Topi S., Godoy F.G., D’Addona A. (2023). Oral Microbiota in Human Health and Disease: A Perspective. Exp. Biol. Med..

[36] Radaic A., Kapila Y.L. (2021). The Oralome and Its Dysbiosis: New Insights into Oral Microbiome-Host Interactions. Comput. Struct. Biotechnol. J..

[37] Xiao L., Zhao F. (2023). Microbial Transmission, Colonisation and Succession: From Pregnancy to Infancy. Gut.

[38] Sedghi L., DiMassa V., Harrington A., Lynch S.V., Kapila Y.L. (2021). The Oral Microbiome: Role of Key Organisms and Complex Networks in Oral Health and Disease. Periodontol. 2000.

[39] Kaan A.M., Kahharova D., Zaura E. (2021). Acquisition and Establishment of the Oral Microbiota. Periodontol. 2000.

[40] Mombelli A. (2018). Microbial Colonization of the Periodontal Pocket and Its Significance for Periodontal Therapy. Periodontol. 2000.

[41] Valm A.M. (2019). The Structure of Dental Plaque Microbial Communities in the Transition from Health to Dental Caries and Periodontal Disease. J. Mol. Biol..

[42] Zhang Y., Wu Y.P., Feng V., Cao G.Z., Feng X.P., Chen X. (2022). Microbiota of Preterm Infant Develops over Time along with the First Teeth Eruption. Front. Microbiol..

[43] Talapko J., Meštrović T., Dmitrović B., Juzbašić M., Matijević T., Bekić S., Erić S., Flam J., Belić D., Petek Erić A. (2023). A Putative Role of Candida Albicans in Promoting Cancer Development: A Current State of Evidence and Proposed Mechanisms. Microorganisms.

[44] Talapko J., Meštrović T., Juzbašić M., Tomas M., Erić S., Horvat Aleksijević L., Bekić S., Schwarz D., Matić S., Neuberg M. (2022). Antimicrobial Peptides—Mechanisms of Action, Antimicrobial Effects and Clinical Applications. Antibiotics.

[45] Sharma N., Bhatia S., Sodhi A.S., Batra N. (2018). Oral Microbiome and Health. AIMS Microbiol..

[46] Zaatout N. (2021). Presence of Non-Oral Bacteria in the Oral Cavity. Arch. Microbiol..

[47] Escobar-Arregocés F., Eras M.A., Bustos A., Suárez-Castillo A., García-Robayo D.A., del Pilar Bernal M. (2024). Characterization of the Oral Microbiota and the Relationship of the Oral Microbiota with the Dental and Periodontal Status in Children and Adolescents with Nonsyndromic Cleft Lip and Palate. Systematic Literature Review and Meta-Analysis. Clin. Oral Investig..

[48] Morrison A.G., Sarkar S., Umar S., Lee S.T.M., Thomas S.M. (2023). The Contribution of the Human Oral Microbiome to Oral Disease: A Review. Microorganisms.

[49] Upadhyay M., Swaroop A., Sinhal V.K., Srivastava A., Garg S.K., Singh V.P., Arora P.K. (2024). Role of Human Oral Microbiome in Diseases. J. Pure Appl. Microbiol..

[50] Li X., Liu Y., Yang X., Li C., Song Z. (2022). The Oral Microbiota: Community Composition, Influencing Factors, Pathogenesis, and Interventions. Front. Microbiol..

[51] Volmer J.G., McRae H., Morrison M. (2023). The Evolving Role of Methanogenic Archaea in Mammalian Microbiomes. Front. Microbiol..

[52] Cheung M.K., Chan J.Y.K., Wong M.C.S., Wong P.Y., Lei P., Cai L., Lan L., Ho W.C.S., Yeung A.C.M., Chan P.K.S. (2022). Determinants and Interactions of Oral Bacterial and Fungal Microbiota in Healthy Chinese Adults. Microbiol. Spectr..

[53] Vallianou N., Kounatidis D., Christodoulatos G.S., Panagopoulos F., Karampela I., Dalamaga M. (2021). Mycobiome and Cancer: What Is the Evidence?. Cancers.

[54] Robinson S., Peterson C.B., Sahasrabhojane P., Ajami N.J., Shelburne S.A., Kontoyiannis D.P., Galloway-Peña J.R. (2020). Observational Cohort Study of Oral Mycobiome and Interkingdom Interactions over the Course of Induction Therapy for Leukemia. mSphere.

[55] Girija A.S.S., Ganesh P.S. (2022). Functional Biomes beyond the Bacteriome in the Oral Ecosystem. Jpn. Dent. Sci. Rev..

[56] Di Spirito F., Di Palo M.P., Folliero V., Cannatà D., Franci G., Martina S., Amato M. (2023). Oral Bacteria, Virus and Fungi in Saliva and Tissue Samples from Adult Subjects with Oral Squamous Cell Carcinoma: An Umbrella Review. Cancers.

[57] Lorini L., Atín C.B., Thavaraj S., Müller-Richter U., Ferranti M.A., Romero J.P., Barba M.S., García-Cuenca A.d.P., García I.B., Bossi P. (2021). Overview of Oral Potentially Malignant Disorders: From Risk Factors to Specific Therapies. Cancers.

[58] Radaic A., Shamir E.R., Jones K., Villa A., Garud N.R., Tward A.D., Kamarajan P., Kapila Y.L. (2023). Specific Oral Microbial Differences in Proteobacteria and Bacteroidetes Are Associated with Distinct Sites When Moving from Healthy Mucosa to Oral Dysplasia-A Microbiome and Gene Profiling Study and Focused Review. Microorganisms.

[59] Pietrobon G., Tagliabue M., Stringa L.M., De Berardinis R., Chu F., Zocchi J., Carlotto E., Chiocca S., Ansarin M. (2021). Leukoplakia in the Oral Cavity and Oral Microbiota: A Comprehensive Review. Cancers.

[60] Herreros-Pomares A., Llorens C., Soriano B., Zhang F., Gallach S., Bagan L., Murillo J., Jantus-Lewintre E., Bagan J. (2021). Oral Microbiome in Proliferative Verrucous Leukoplakia Exhibits Loss of Diversity and Enrichment of Pathogens. Oral Oncol..

[61] Chattopadhyay I., Verma M., Panda M. (2019). Role of Oral Microbiome Signatures in Diagnosis and Prognosis of Oral Cancer. Technol. Cancer Res. Treat..

[62] Shih Y.H., Wang T.H., Shieh T.M., Tseng Y.H. (2019). Oral Submucous Fibrosis: A Review on Etiopathogenesis, Diagnosis, and Therapy. Int. J. Mol. Sci..

[63] Chocolatewala N., Chaturvedi P., Desale R. (2010). The Role of Bacteria in Oral Cancer. Indian J. Med. Paediatr. Oncol..

[64] Kurtzman G.M., Horowitz R.A., Johnson R., Prestiano R.A., Klein B.I. (2022). The Systemic Oral Health Connection: Biofilms. Medicine.

[65] Vestby L.K., Grønseth T., Simm R., Nesse L.L. (2020). Bacterial Biofilm and Its Role in the Pathogenesis of Disease. Antibiotics.

[66] Choi E., Murray B., Choi S. (2023). Biofilm and Cancer: Interactions and Future Directions for Cancer Therapy. Int. J. Mol. Sci..

[67] Talapko J., Škrlec I. (2020). The Principles, Mechanisms, and Benefits of Unconventional Agents in the Treatment of Biofilm Infection. Pharmaceuticals.

[68] Aleksijević L.H., Aleksijević M., Škrlec I., Šram M., Talapko J. (2022). *Porphyromonas gingivalis* Virulence Factors and Clinical Significance in Periodontal Disease and Coronary Artery Diseases. Pathogens.

[69] Sterzenbach T., Helbig R., Hannig C., Hannig M. (2020). Bioadhesion in the Oral Cavity and Approaches for Biofilm Management by Surface Modifications. Clin. Oral Investig..

[70] Zhao A., Sun J., Liu Y. (2023). Understanding Bacterial Biofilms: From Definition to Treatment Strategies. Front. Cell. Infect. Microbiol..

[71] Pignatelli P., Nuccio F., Piattelli A., Curia M.C. (2023). The Role of *Fusobacterium nucleatum* in Oral and Colorectal Carcinogenesis. Microorganisms.

[72] Zepeda-Rivera M., Minot S.S., Bouzek H., Wu H., Blanco-Míguez A., Manghi P., Jones D.S., LaCourse K.D., Wu Y., McMahon E.F. (2024). A Distinct *Fusobacterium nucleatum* Clade Dominates the Colorectal Cancer Niche. Nature.

[73] Gerits E., Verstraeten N., Michiels J. (2017). New Approaches to Combat *Porphyromonas gingivalis* Biofilms. J. Oral Microbiol..

[74] Chenicheri S., Usha R., Ramachandran R., Thomas V., Wood A. (2017). Insight into Oral Biofilm: Primary, Secondary and Residual Caries and Phyto-Challenged Solutions. Open Dent. J..

[75] Sauer K., Stoodley P., Goeres D.M., Hall-Stoodley L., Burmølle M., Stewart P.S., Bjarnsholt T. (2022). The Biofilm Life Cycle: Expanding the Conceptual Model of Biofilm Formation. Nat. Rev. Microbiol..

[76] Zhao X., Yu Z., Ding T. (2020). Quorum-Sensing Regulation of Antimicrobial Resistance in Bacteria. Microorganisms.

[77] Balducci E., Papi F., Capialbi D.E., Del Bino L. (2023). Polysaccharides’ Structures and Functions in Biofilm Architecture of Antimicrobial-Resistant (AMR) Pathogens. Int. J. Mol. Sci..

[78] Karygianni L., Ren Z., Koo H., Thurnheer T. (2020). Biofilm Matrixome: Extracellular Components in Structured Microbial Communities. Trends Microbiol..

[79] Rumbaugh K.P., Sauer K. (2020). Biofilm Dispersion. Nat. Rev. Microbiol..

[80] Sharma S., Mohler J., Mahajan S.D., Schwartz S.A., Bruggemann L., Aalinkeel R. (2023). Microbial Biofilm: A Review on Formation, Infection, Antibiotic Resistance, Control Measures, and Innovative Treatment. Microorganisms.

[81] Che S., Yan Z., Feng Y., Zhao H. (2024). Unveiling the Intratumoral Microbiota within Cancer Landscapes. iScience.

[82] Kay J., Thadhani E., Samson L., Engelward B. (2019). Inflammation-Induced DNA Damage, Mutations and Cancer. DNA Repair.

[83] Deng Y., Liu S.Y., Chua S.L., Khoo B.L. (2021). The Effects of Biofilms on Tumor Progression in a 3D Cancer-Biofilm Microfluidic Model. Biosens. Bioelectron..

[84] Ciernikova S., Sevcikova A., Stevurkova V., Mego M. (2022). Tumor Microbiome—An Integral Part of the Tumor Microenvironment. Front. Oncol..

[85] Min Z., Yang L., Hu Y., Huang R. (2023). Oral Microbiota Dysbiosis Accelerates the Development and Onset of Mucositis and Oral Ulcers. Front. Microbiol..

[86] Spatafora G., Li Y., He X., Cowan A., Tanner A.C.R. (2024). The Evolving Microbiome of Dental Caries. Microorganisms.

[87] Lavoro A., Cultrera G., Gattuso G., Lombardo C., Falzone L., Saverio C., Libra M., Salmeri M. (2024). Role of Oral Microbiota Dysbiosis in the Development and Progression of Oral Lichen Planus. J. Pers. Med..

[88] Siddiqui R., Badran Z., Boghossian A., Alharbi A.M., Alfahemi H., Khan N.A. (2023). The Increasing Importance of the Oral Microbiome in Periodontal Health and Disease. Future Sci. OA.

[89] la Rosa G.R.M., Gattuso G., Pedullà E., Rapisarda E., Nicolosi D., Salmeri M. (2020). Association of Oral Dysbiosis with Oral Cancer Development. Oncol. Lett..

[90] Sarkar P., Malik S., Laha S., Das S., Bunk S., Ray J.G., Chatterjee R., Saha A. (2021). Dysbiosis of Oral Microbiota During Oral Squamous Cell Carcinoma Development. Front. Oncol..

[91] Yang J., He P., Zhou M., Li S., Zhang J., Tao X., Wang A., Wu X. (2022). Variations in Oral Microbiome and Its Predictive Functions between Tumorous and Healthy Individuals. J. Med. Microbiol..

[92] Delaney C., Veena C.L.R., Butcher M.C., McLean W., Shaban S.M.A., Nile C.J., Ramage G. (2023). Limitations of Using 16S RRNA Microbiome Sequencing to Predict Oral Squamous Cell Carcinoma. APMIS.

[93] Su Mun L., Wye Lum S., Kong Yuiin Sze G., Hock Yoong C., Ching Yung K., Kah Lok L., Gopinath D. (2021). Association of Microbiome with Oral Squamous Cell Carcinoma: A Systematic Review of the Metagenomic Studies. Int. J. Environ. Res. Public Health.

[94] Sukmana B.I., Saleh R.O., Najim M.A., AL-Ghamdi H.S., Achmad H., Al-Hamdani M.M., Taher A.A.Y., Alsalamy A., Khaledi M., Javadi K. (2024). Oral Microbiota and Oral Squamous Cell Carcinoma: A Review of Their Relation and Carcinogenic Mechanisms. Front. Oncol..

[95] Vyhnalova T., Danek Z., Gachova D., Linhartova P.B. (2021). The Role of the Oral Microbiota in the Etiopathogenesis of Oral Squamous Cell Carcinoma. Microorganisms.

[96] Li Q., Hu Y., Zhou X., Liu S., Han Q., Cheng L. (2020). Role of Oral Bacteria in the Development of Oral Squamous Cell Carcinoma. Cancers.

[97] Ye C., Liu X., Liu Z., Pan C., Zhang X., Zhao Z., Sun H. (2024). *Fusobacterium nucleatum* in Tumors: From Tumorigenesis to Tumor Metastasis and Tumor Resistance. Cancer Biol. Ther..

[98] Alon-Maimon T., Mandelboim O., Bachrach G. (2022). *Fusobacterium nucleatum* and Cancer. Periodontol. 2000.

[99] McIlvanna E., Linden G.J., Craig S.G., Lundy F.T., James J.A. (2021). *Fusobacterium nucleatum* and Oral Cancer: A Critical Review. BMC Cancer.

[100] Chen Y., Huang Z., Tang Z., Huang Y., Huang M., Liu H., Ziebolz D., Schmalz G., Jia B., Zhao J. (2022). More Than Just a Periodontal Pathogen—The Research Progress on Fusobacterium Nucleatum. Front. Cell. Infect. Microbiol..

[101] Yang Y.L., Yang F., Huang Z.Q., Li Y.Y., Shi H.Y., Sun Q., Ma Y., Wang Y., Zhang Y., Yang S. (2023). T Cells, NK Cells, and Tumor-Associated Macrophages in Cancer Immunotherapy and the Current State of the Art of Drug Delivery Systems. Front. Immunol..

[102] Sakamoto Y., Mima K., Ishimoto T., Ogata Y., Imai K., Miyamoto Y., Akiyama T., Daitoku N., Hiyoshi Y., Iwatsuki M. (2021). Relationship between *Fusobacterium nucleatum* and Antitumor Immunity in Colorectal Cancer Liver Metastasis. Cancer Sci..

[103] Groeger S., Zhou Y., Ruf S., Meyle J. (2022). Pathogenic Mechanisms of *Fusobacterium nucleatum* on Oral Epithelial Cells. Front. Oral Health.

[104] Fan Z., Tang P., Li C., Yang Q., Xu Y., Su C., Li L. (2022). *Fusobacterium nucleatum* and Its Associated Systemic Diseases: Epidemiologic Studies and Possible Mechanisms. J. Oral Microbiol..

[105] Wang S., Liu Y., Li J., Zhao L., Yan W., Lin B., Guo X., Wei Y. (2021). *Fusobacterium nucleatum* Acts as a Pro-Carcinogenic Bacterium in Colorectal Cancer: From Association to Causality. Front. cell Dev. Biol..

[106] Liang B., Wu C., Wang C., Sun W., Chen W., Hu X., Liu N., Xing D. (2022). New Insights into Bacterial Mechanisms and Potential Intestinal Epithelial Cell Therapeutic Targets of Inflammatory Bowel Disease. Front. Microbiol..

[107] Sun J., Tang Q., Yu S., Xie M., Zheng W., Chen G., Yin Y., Huang X., Wo K., Lei H. (2023). *F. Nucleatum* Facilitates Oral Squamous Cell Carcinoma Progression via GLUT1-Driven Lactate Production. EBioMedicine.

[108] Sezgin E., Terlemez G., Bozkurt B., Bengi G., Akpinar H., Büyüktorun İ. (2022). Quantitative Real-Time PCR Analysis of Bacterial Biomarkers Enable Fast and Accurate Monitoring in Inflammatory Bowel Disease. PeerJ.

[109] Xue X., Li R., Chen Z., Li G., Liu B., Guo S., Yue Q., Yang S., Xie L., Zhang Y. (2023). The Role of the Symbiotic Microecosystem in Cancer: Gut Microbiota, Metabolome, and Host Immunome. Front. Immunol..

[110] Tuominen H., Rautava J. (2021). Oral Microbiota and Cancer Development. Pathobiology.

[111] Bi R., Yang Y., Liao H., Ji G., Ma Y., Cai L., Li J., Yang J., Sun M., Liang J. (2023). *Porphyromonas gingivalis* Induces an Inflammatory Response via the CGAS-STING Signaling Pathway in a Periodontitis Mouse Model. Front. Microbiol..

[112] Yi M., Li T., Niu M., Zhang H., Wu Y., Wu K., Dai Z. (2024). Targeting Cytokine and Chemokine Signaling Pathways for Cancer Therapy. Signal Transduct. Target. Ther..

[113] Chen W.A., Dou Y., Fletcher H.M., Boskovic D.S. (2023). Local and Systemic Effects of *Porphyromonas gingivalis* Infection. Microorganisms.

[114] Kumar S., Jeong Y., Ashraf M.U., Bae Y.S. (2019). Dendritic Cell-Mediated Th2 Immunity and Immune Disorders. Int. J. Mol. Sci..

[115] Muñoz-Medel M., Pinto M.P., Goralsky L., Cáceres M., Villarroel-Espíndola F., Manque P., Pinto A., Garcia-Bloj B., de Mayo T., Godoy J.A. (2024). Porphyromonas Gingivalis, a Bridge between Oral Health and Immune Evasion in Gastric Cancer. Front. Oncol..

[116] Li N., Collyer C.A. (2011). Gingipains from *Porphyromonas gingivalis*—Complex Domain Structures Confer Diverse Functions. Eur. J. Microbiol. Immunol..

[117] Mu W., Jia Y., Chen X., Li H., Wang Z., Cheng B. (2020). Intracellular *Porphyromonas gingivalis* Promotes the Proliferation of Colorectal Cancer Cells via the MAPK/ERK Signaling Pathway. Front. Cell. Infect. Microbiol..

[118] Shahoumi L.A., Saleh M.H.A., Meghil M.M. (2023). Virulence Factors of the Periodontal Pathogens: Tools to Evade the Host Immune Response and Promote Carcinogenesis. Microorganisms.

[119] Mei F., Xie M., Huang X., Long Y., Lu X., Wang X., Chen L. (2020). *Porphyromonas gingivalis* and Its Systemic Impact: Current Status. Pathogens.

[120] Yáñez L., Soto C., Tapia H., Pacheco M., Tapia J., Osses G., Salinas D., Rojas-Celis V., Hoare A., Quest A.F.G. (2024). Co-Culture of *P. Gingivalis* and *F. Nucleatum* Synergistically Elevates IL-6 Expression via TLR4 Signaling in Oral Keratinocytes. Int. J. Mol. Sci..

[121] Chopra A., Bhat S.G., Sivaraman K. (2020). *Porphyromonas gingivalis* Adopts Intricate and Unique Molecular Mechanisms to Survive and Persist within the Host: A Critical Update. J. Oral Microbiol..

[122] Chan K.T., Song X., Shen L., Liu N., Zhou X., Cheng L., Chen J. (2023). Nisin and Its Application in Oral Diseases. J. Funct. Foods.

[123] Barranca-Enríquez A., Romo-González T. (2022). Your Health Is in Your Mouth: A Comprehensive View to Promote General Wellness. Front. Oral Health.

[124] Brookes Z., McGrath C., McCullough M. (2023). Antimicrobial Mouthwashes: An Overview of Mechanisms-What Do We Still Need to Know?. Int. Dent. J..

[125] Ciani L., Libonati A., Dri M., Pomella S., Campanella V., Barillari G. (2024). About a Possible Impact of Endodontic Infections by *Fusobacterium nucleatum* or *Porphyromonas gingivalis* on Oral Carcinogenesis: A Literature Overview. Int. J. Mol. Sci..

[126] Talapko J., Juzbašić M., Meštrović T., Matijević T., Mesarić D., Katalinić D., Erić S., Milostić-Srb A., Flam J., Škrlec I. (2024). *Aggregatibacter actinomycetemcomitans*: From the Oral Cavity to the Heart Valves. Microorganisms.

[127] Baima G., Minoli M., Michaud D.S., Aimetti M., Sanz M., Loos B.G., Romandini M. (2023). Periodontitis and Risk of Cancer: Mechanistic Evidence. Periodontol. 2000.

[128] Barsouk A., Aluru J.S., Rawla P., Saginala K., Barsouk A. (2023). Epidemiology, Risk Factors, and Prevention of Head and Neck Squamous Cell Carcinoma. Med. Sci..

[129] Nokovitch L., Maquet C., Crampon F., Taihi I., Roussel L.M., Obongo R., Virard F., Fervers B., Deneuve S. (2023). Oral Cavity Squamous Cell Carcinoma Risk Factors: State of the Art. J. Clin. Med..

[130] Chu D., Liu T., Yao Y. (2023). Implications of Viral Infections and Oncogenesis in Uterine Cervical Carcinoma Etiology and Pathogenesis. Front. Microbiol..

[131] Gupta S.L., Basu S., Soni V., Jaiswal R.K. (2022). Immunotherapy: An Alternative Promising Therapeutic Approach against Cancers. Mol. Biol. Rep..

[132] Lechner M., Liu J., Masterson L., Fenton T.R. (2022). HPV-Associated Oropharyngeal Cancer: Epidemiology, Molecular Biology and Clinical Management. Nat. Rev. Clin. Oncol..

[133] Egawa N. (2023). Papillomaviruses and Cancer: Commonalities and Differences in HPV Carcinogenesis at Different Sites of the Body. Int. J. Clin. Oncol..

[134] Gillison M.L., Akagi K., Xiao W., Jiang B., Pickard R.K.L., Li J., Swanson B.J., Agrawal A.D., Zucker M., Stache-Crain B. (2019). Human Papillomavirus and the Landscape of Secondary Genetic Alterations in Oral Cancers. Genome Res..

[135] Yeo-Teh N.S.L., Ito Y., Jha S. (2018). High-Risk Human Papillomaviral Oncogenes E6 and E7 Target Key Cellular Pathways to Achieve Oncogenesis. Int. J. Mol. Sci..

[136] Graham S.V. (2017). The Human Papillomavirus Replication Cycle, and Its Links to Cancer Progression: A Comprehensive Review. Clin. Sci..

[137] Berman T.A., Schiller J.T. (2017). Human Papillomavirus in Cervical Cancer and Oropharyngeal Cancer: One Cause, Two Diseases. Cancer.

[138] Chihu-Amparan L., Pedroza-Saavedra A., Gutierrez-Xicotencatl L. (2023). The Immune Response Generated against HPV Infection in Men and Its Implications in the Diagnosis of Cancer. Microorganisms.

[139] Moody C.A. (2022). Regulation of the Innate Immune Response during the Human Papillomavirus Life Cycle. Viruses.

[140] Jiang Y., Tsoi L.C., Billi A.C., Ward N.L., Harms P.W., Zeng C., Maverakis E., Michelle Kahlenberg J., Gudjonsson J.E. (2020). Cytokinocytes: The Diverse Contribution of Keratinocytes to Immune Responses in Skin. JCI Insight.

[141] van Bockel D., Kelleher A. (2023). The Crossroads: Divergent Roles of Virus-Specific CD4+ T Lymphocytes in Determining the Outcome for Human Papillomavirus Infection. Immunol. Cell Biol..

[142] Lechien J.R., Seminerio I., Descamps G., Mat Q., Mouawad F., Hans S., Julieron M., Dequanter D., Vanderhaegen T., Journe F. (2019). Impact of HPV Infection on the Immune System in Oropharyngeal and Non-Oropharyngeal Squamous Cell Carcinoma: A Systematic Review. Cells.

[143] Damania B., Kenney S.C., Raab-Traub N. (2022). Epstein-Barr Virus: Biology and Clinical Disease. Cell.

[144] Wang L., Ning S. (2021). New Look of EBV LMP1 Signaling Landscape. Cancers.

[145] Cereser B., Jansen M., Austin E., Elia G., McFarlane T., van Deurzen C.H.M., Sieuwerts A.M., Daidone M.G., Tadrous P.J., Wright N.A. (2018). Analysis of Clonal Expansions through the Normal and Premalignant Human Breast Epithelium Reveals the Presence of Luminal Stem Cells. J. Pathol..

[146] Rex V., Zargari R., Stempel M., Halle S., Brinkmann M.M. (2023). The Innate and T-Cell Mediated Immune Response during Acute and Chronic Gammaherpesvirus Infection. Front. Cell. Infect. Microbiol..

[147] Silva J.d.M., Alves C.E.d.C., Pontes G.S. (2024). Epstein-Barr Virus: The Mastermind of Immune Chaos. Front. Immunol..

[148] Chakravorty S., Afzali B., Kazemian M. (2022). EBV-Associated Diseases: Current Therapeutics and Emerging Technologies. Front. Immunol..

[149] Li W., Duan X., Chen X., Zhan M., Peng H., Meng Y., Li X., Li X.Y., Pang G., Dou X. (2023). Immunotherapeutic Approaches in EBV-Associated Nasopharyngeal Carcinoma. Front. Immunol..

[150] Berwanger A., Stein S.C., Kany A.M., Gartner M., Loretz B., Lehr C.M., Hirsch A.K.H., Schulz T.F., Empting M. (2023). Disrupting Kaposi’s Sarcoma-Associated Herpesvirus (KSHV) Latent Replication with a Small Molecule Inhibitor. J. Med. Chem..

[151] Reddy N.A., Mays S.R., Pacha O. (2019). Kaposi’s Sarcoma in the Immunosuppressed. J. Immunother. Precis. Oncol..

[152] Lopes A.D., Marinho P.D., Medeiros L.D., de Paula V.S. (2022). Human Gammaherpesvirus 8 Oncogenes Associated with Kaposi’s Sarcoma. Int. J. Mol. Sci..

[153] Angius F., Ingianni A., Pompei R. (2020). Human Herpesvirus 8 and Host-Cell Interaction: Long-Lasting Physiological Modifications, Inflammation and Related Chronic Diseases. Microorganisms.

[154] Schulz T.F., Cesarman E. (2015). Kaposi Sarcoma-Associated Herpesvirus: Mechanisms of Oncogenesis. Curr. Opin. Virol..

[155] Tornesello A.L., Tagliamonte M., Buonaguro F.M., Tornesello M.L., Buonaguro L. (2022). Virus-like Particles as Preventive and Therapeutic Cancer Vaccines. Vaccines.

[156] Kamel M.S., Munds R.A., Verma M.S. (2023). The Quest for Immunity: Exploring Human Herpesviruses as Vaccine Vectors. Int. J. Mol. Sci..

[157] Mamilos A., Lein A., Winter L., Ettl T., Künzel J., Reichert T.E., Spanier G., Brochhausen C. (2023). Tumor Immune Microenvironment Heterogeneity at the Invasion Front and Tumor Center in Oral Squamous Cell Carcinoma as a Perspective of Managing This Cancer Entity. J. Clin. Med..

[158] Ferris R.L., Blumenschein G., Fayette J., Guigay J., Colevas A.D., Licitra L., Harrington K., Kasper S., Vokes E.E., Even C. (2016). Nivolumab for Recurrent Squamous-Cell Carcinoma of the Head and Neck. N. Engl. J. Med..

[159] Borsetto D., Tomasoni M., Payne K., Polesel J., Deganello A., Bossi P., Tysome J.R., Masterson L., Tirelli G., Tofanelli M. (2021). Prognostic Significance of CD4+ and CD8+ Tumor-Infiltrating Lymphocytes in Head and Neck Squamous Cell Carcinoma: A Meta-Analysis. Cancers.

[160] Wang J., Tian S., Sun J., Zhang J., Lin L., Hu C. (2020). The Presence of Tumour-Infiltrating Lymphocytes (TILs) and the Ratios between Different Subsets Serve as Prognostic Factors in Advanced Hypopharyngeal Squamous Cell Carcinoma. BMC Cancer.

[161] Brierly G., Celentano A., Breik O., Moslemivayeghan E., Patini R., McCullough M., Yap T. (2023). Tumour Necrosis Factor Alpha (TNF-α) and Oral Squamous Cell Carcinoma. Cancers.

[162] Laliberté C., Ng N., Eymael D., Higgins K., Ali A., Kiss A., Bradley G., Magalhaes M.A.O. (2021). Characterization of Oral Squamous Cell Carcinoma Associated Inflammation: A Pilot Study. Front. Oral Health.

[163] de Visser K.E., Joyce J.A. (2023). The Evolving Tumor Microenvironment: From Cancer Initiation to Metastatic Outgrowth. Cancer Cell.

[164] Salemme V., Centonze G., Cavallo F., Defilippi P., Conti L. (2021). The Crosstalk Between Tumor Cells and the Immune Microenvironment in Breast Cancer: Implications for Immunotherapy. Front. Oncol..

[165] Burtness B., Harrington K.J., Greil R., Soulières D., Tahara M., de Castro G., Psyrri A., Basté N., Neupane P., Bratland Å. (2019). Pembrolizumab Alone or with Chemotherapy versus Cetuximab with Chemotherapy for Recurrent or Metastatic Squamous Cell Carcinoma of the Head and Neck (KEYNOTE-048): A Randomised, Open-Label, Phase 3 Study. Lancet.

[166] Wang H.Q., Fu R., Man Q.W., Yang G., Liu B., Bu L.L. (2023). Advances in CAR-T Cell Therapy in Head and Neck Squamous Cell Carcinoma. J. Clin. Med..

[167] Cheng W., Li F., Gao Y., Yang R. (2024). Fungi and Tumors: The Role of Fungi in Tumorigenesis (Review). Int. J. Oncol..

[168] Ghannoum M.A., Jurevic R.J., Mukherjee P.K., Cui F., Sikaroodi M., Naqvi A., Gillevet P.M. (2010). Characterization of the Oral Fungal Microbiome (Mycobiome) in Healthy Individuals. PLoS Pathog..

[169] Pires R.H., Martinez L.R., Mendes-Giannini M.J.S., Stoianoff M.A.R. (2021). Editorial: Pathogenesis of Fungal Biofilms in Different Environmental Conditions and Clinical Outcomes. Front. Cell. Infect. Microbiol..

[170] Cui L., Morris A., Ghedin E. (2013). The Human Mycobiome in Health and Disease. Genome Med..

[171] Wang X., Wu S., Wu W., Zhang W., Li L., Liu Q., Yan Z. (2023). Candida Albicans Promotes Oral Cancer via IL-17A/IL-17RA-Macrophage Axis. MBio.

[172] Ladjevac N., Milovanovic M., Jevtovic A., Arsenijevic D., Stojanovic B., Dimitrijevic Stojanovic M., Stojanovic B., Arsenijevic N., Arsenijevic A., Milovanovic J. (2023). The Role of IL-17 in the Pathogenesis of Oral Squamous Cell Carcinoma. Int. J. Mol. Sci..

[173] Bilal H., Khan M.N., Khan S., Fang W., Chang W., Yin B., Song N.J., Liu Z., Zhang D., Yao F. (2023). Risk of Candidiasis Associated with Interleukin-17 Inhibitors: Implications and Management. Mycology.

[174] Hwang G. (2022). In It Together: Candida-Bacterial Oral Biofilms and Therapeutic Strategies. Environ. Microbiol. Rep..

[175] Du Q., Ren B., Zhou X., Zhang L., Xu X. (2022). Cross-Kingdom Interaction between Candida Albicans and Oral Bacteria. Front. Microbiol..

[176] de Jongh C.A., Bikker F.J., de Vries T.J., Werner A., Gibbs S., Krom B.P. (2023). *Porphyromonas gingivalis* Interaction with Candida Albicans Allows for Aerobic Escape, Virulence and Adherence. Biofilm.

[177] Martorano-Fernandes L., Goodwine J.S., Ricomini-Filho A.P., Nobile C.J., Del Bel Cury A.A. (2023). Candida Albicans Adhesins Als1 and Hwp1 Modulate Interactions with Streptococcus Mutans. Microorganisms.

[178] Ciurea C.N., Kosovski I.-B., Mare A.D., Toma F., Pintea-Simon I.A., Man A. (2020). Candida and Candidiasis—Opportunism Versus Pathogenicity: A Review of the Virulence Traits. Microorganisms.

[179] Bartnicka D., Gonzalez-Gonzalez M., Sykut J., Koziel J., Ciaston I., Adamowicz K., Bras G., Zawrotniak M., Karkowska-Kuleta J., Satala D. (2020). Candida Albicans Shields the Periodontal Killer *Porphyromonas gingivalis* from Recognition by the Host Immune System and Supports the Bacterial Infection of Gingival Tissue. Int. J. Mol. Sci..

[180] Mukherjee P.K., Wang H., Retuerto M., Zhang H., Burkey B., Ghannoum M.A., Eng C. (2017). Bacteriome and Mycobiome Associations in Oral Tongue Cancer. Oncotarget.

[181] Di Cosola M., Cazzolla A.P., Charitos I.A., Ballini A., Inchingolo F., Santacroce L. (2021). Candida Albicans and Oral Carcinogenesis. A Brief Review. J. Fungi.

[182] Ayuningtyas N.F., Mahdani F.Y., Pasaribu T.A.S., Chalim M., Ayna V.K.P., Santosh A.B.R., Santacroce L., Surboyo M.D.C. (2022). Role of Candida Albicans in Oral Carcinogenesis. Pathophysiology.

[183] Talapko J., Juzbašić M., Matijević T., Pustijanac E., Bekić S., Kotris I., Škrlec I. (2021). Candida Albicans-The Virulence Factors and Clinical Manifestations of Infection. J. Fungi.

[184] Ilkhanizadeh-Qomi M., Nejatbakhsh S., Jahanshiri Z., Razzaghi-Abyaneh M. (2020). Aspartyl Proteinase and Phospholipase Activities of Candida Albicans Isolated From Oropharyngeal Candidiasis in Head and Neck Cancer Patients. Jundishapur. J. Microbiol..

[185] Mogavero S., Höfs S., Lauer A.N., Müller R., Brunke S., Allert S., Gerwien F., Groth S., Dolk E., Wilson D. (2022). Candidalysin Is the Hemolytic Factor of Candida Albicans. Toxins.

[186] Richardson J.P., Brown R., Kichik N., Lee S., Priest E., Mogavero S., Maufrais C., Wickramasinghe D.N., Tsavou A., Kotowicz N.K. (2022). Candidalysins Are a New Family of Cytolytic Fungal Peptide Toxins. MBio.

[187] Nikou S.A., Zhou C., Griffiths J.S., Kotowicz N.K., Coleman B.M., Green M.J., Moyes D.L., Gaffen S.L., Naglik J.R., Parker P.J. (2022). The Candida Albicans Toxin Candidalysin Mediates Distinct Epithelial Inflammatory Responses through P38 and EGFR-ERK Pathways. Sci. Signal..

[188] Zhao H., Wu L., Yan G., Chen Y., Zhou M., Wu Y., Li Y. (2021). Inflammation and Tumor Progression: Signaling Pathways and Targeted Intervention. Signal Transduct. Target. Ther..

[189] Engku Nasrullah Satiman E.A.F., Ahmad H., Ramzi A.B., Abdul Wahab R., Kaderi M.A., Wan Harun W.H.A., Dashper S., McCullough M., Arzmi M.H. (2020). The Role of Candida Albicans Candidalysin ECE1 Gene in Oral Carcinogenesis. J. Oral Pathol. Med..

[190] Vadovics M., Ho J., Igaz N., Alföldi R., Rakk D., Veres E., Szücs B., Horváth M., Tóth R., Szücs A. (2021). Candida Albicans Enhances the Progression of Oral Squamous Cell Carcinoma In Vitro and In Vivo. MBio.

[191] Khanam A., Hithamani G., Naveen J., Pradeep S.R., Barman S., Srinivasan K. (2023). Management of Invasive Infections in Diabetes Mellitus: A Comprehensive Review. Biologics.

[192] Thompson A., Orr S.J. (2018). Emerging IL-12 Family Cytokines in the Fight against Fungal Infections. Cytokine.

[193] Hosseini K., Ahangari H., Chapeland-Leclerc F., Ruprich-Robert G., Tarhriz V., Dilmaghani A. (2022). Role of Fungal Infections in Carcinogenesis and Cancer Development: A Literature Review. Adv. Pharm. Bull..

[194] Dwivedi P.P., Mallya S., Dongari-Bagtzoglou A. (2009). A Novel Immunocompetent Murine Model for Candida Albicans-Promoted Oral Epithelial Dysplasia. Med. Mycol..

[195] Fadhil Ali Malaa S., Abd Ali Abd Aun Jwad B., Khalis Al-Masoudi H. (2022). Assessment of Entamoeba Gingivalis and Trichomonas Tenax in Patients with Chronic Diseases and Its Correlation with Some Risk Factors. Arch. Razi Inst..

[196] Sun J., Tang Q., Yu S., Xie M., Xie Y., Chen G., Chen L. (2020). Role of the oral microbiota in cancer evolution and progression. Cancer Med..

